# The Book World of Medicine and Science

**Published:** 1895-06-15

**Authors:** 


### The Book World of Medicine and Science.

Growing Children and Awkward Walking. By Thomas William Nunn, F.R.C.S.Eng., Consulting Surgeon to the Middlesex Hospital, &c. (London: Kegan Paul, Trench, Trübner, and Co., Limited, Paternoster House, Charing Cross Road.)

Englishwomen can vie with those of any other nation in features, surpass them in complexion, and in figure, but it is admitted even by admiring critics that they lack grace of movement. The same is doubtless true of Englishmen, and one cannot but wonder why the most health-loving race in the world should rank among the most awkward. Mr. Nunn shows that a very large proportion of us have limbs of unequal length, which produces often an ungainly gait, and occasionally in the involuntary effort to avoid irregularity of step, a lateral curvature of the spine. Occasionally too, diseases which have no apparent connection with the feet may interfere with the nutrition of the muscles used in walking, and thus affect the step, leading sometimes to bunions, corns, and other evils, for which most people seek a local rather than a constitutional cure. Yet to be effective a cure must begin with the centres rather than the extremities, and, moreover, the ailment must be attended to as soon as it is perceived before a bad habit of walking has produced absolute distortions Mr. Nunn suggests some useful gymnastics for children afflicted with defects of gait, but the chief value of his book lies in bringing before parents the risks that may spring from apparently slight and remote causes, and the importance of paying attention to the first symptoms of awkwardness in walking.

Text-book of Diseases of the Kidneys and Urinary Organs. By Professor Dr. Paul Fürbringer. Translated from the German, with annotations, by W. H. Gilbert, M.D. (In two volumes. Vol I., pp. 194. Price 7s. 6d. London, H. K. Lewis. 1895.)

Those who desire a fairly complete work on diseases of the kidney as seen from a German standpoint will find this book one of considerable interest. In a series of chapters on albuminuria, hæmaturia, hæmoglobinuria, casts, &c., a description is given of the more prominent symptoms of the various diseases of the kidneys, and the clinical significance of each is fully discussed. After speaking of, so-called, physiological albuminuria, it is pointed out that the question whether such a condition exists is largely a matter of the meaning of the term. Fortunately for the practical utility of albuminuria, as a symptom of disease, complicated tests are necessary to prove the presence of albumin in normal urines. Hence the clinical advantage of the rougher tests. "Practitioners should adopt as much as possible the old and reliable procedures of testing for albumin, should not trouble themselves about small unimportant hazes, but should only reckon with decided deposits, which even at first sight show their importance." A full and careful account is given of uræmia, and the many theories of its causation having been discussed, the conclusion is arrived at that "what we nowadays term uræmia is a clinical collective name, and that an attempt to establish one sole cause as an explanation of all forms is, according to the extent of our present science, futile." It is an overloading of the blood with urinary substances, ensuing from a pathological disproportion between formation and excretion, and therefore a form of auto intoxication, but whether urea, potash salts, or alkaloids play the chief part cannot yet be decided. The major part of the work is devoted to a consideration of the special diseases of the kidney, dividing them into conditions arising (*a*) from circulatory disturbances, and (*b*) from inflammation. Under the latter heading and its results come the mass of the diseases special to the kidney. The symptoms and the pathology of all these are well described, and whenever the author speaks in the first person, giving his own conclusions and the results of his own experience, the matter is interesting enough. There can be no doubt, however, that the very frequent references to the, often contradictory, opinions of others detracts considerably from the readability of the book, much as it may add to its utility as a work of reference.

### MAGAZINES OF THE MONTH.

### "The Humanitarian."

Social questions of somewhat vexed an order we always find discussed in the "Humanitarian" in a grave and thoughtful manner. Whether the same questions touched in a lighter manner in fiction are of the same value is an unsolved problem yet. It is certain that the short stories in the pages of this magazine always go to the heart of the matter, such matter being invariably founded on social and moral questions. In the present instance, the subject is "The Ban of Birth." "S x Prejudice and Woman's Progress" is discussed with some partiality, yet with a great deal of reason on its side, as is also "The Seat of Authority in Morality," by Powis Houet.

